# A case report of fatal anaphylaxis on first exposure to rasburicase just before lymphoma treatment

**DOI:** 10.1186/s13223-024-00920-9

**Published:** 2024-10-26

**Authors:** Yoshikazu Utsu, Natsuho Kaneda, Makio Kawakami, Shin-ichi Masuda, Hironori Arai, Sonoko Shimoji, Rena Matsumoto, Takafumi Tsushima, Kazusuke Tanaka, Kosuke Matsuo, Chiharu Kimeda, Shiho Konno, Nobuyuki Aotsuka

**Affiliations:** 1 Department of Medical Oncology, Japanese Red Cross Narita Hospital, 90-1, Iida-Cho, Narita, 286-8583 Japan; 2 Department of Hematology and Oncology, Japanese Red Cross Narita Hospital, Narita, Japan; 3 Department of Pathology, Japanese Red Cross Narita Hospital, Narita, Japan

**Keywords:** Rasburicase, Anaphylaxis, Allergy, Asthma, Initial administration, Lymphoma, Shock, Fatal, Death

## Abstract

**Background:**

Rasburicase, a recombinant urate oxidase enzyme, has potent efficacy in controlling uric acid and is widely used to prevent tumor lysis syndrome in high-risk patients owing to its low toxicity profile. However, it has been associated with a risk of anaphylaxis, especially on re-exposure, owing to its immunogenic potential.

**Case presentation:**

A 71-year-old Japanese female diagnosed with diffuse large B cell lymphoma with a large tumor burden experienced anaphylactic shock leading to death upon initial administration of rasburicase. The pre-and postmortem examination revealed that the cause of death was a cascade of events starting with anaphylaxis-induced distributive shock leading to obstructive shock due to the collapse of the heart, which was compressed by the post-mediastinal tumor. This was further compounded by massive bleeding from the tumor and tension hemothorax, resulting in circulatory collapse.

**Conclusions:**

Although extremely rare, rasburicase can cause fatal anaphylaxis, even on first exposure.

## Background

Rasburicase, a recombinant form of urate oxidase produced by introducing and expressing the uricase gene derived from *Aspergillus flavus* in *Saccharomyces cerevisiae* (*S. cerevisiae*) strains [1], has demonstrated potent efficacy in controlling uric acid in several trials of pediatric and adult patients with hematologic malignancy [2, 3]. Although rasburicase is contraindicated in patients with glucose-6-phosphate dehydrogenase deficiency because of the risk of hemolysis [4, 5], its safety profile has been demonstrated in clinical trials of patients without glucose-6-phosphate dehydrogenase deficiency. Currently, with the accumulation of experience on its use, rasburicase has become widely recommended and is used as a prophylactic agent for high-risk patients (and some intermediate-risk patients) with tumor lysis syndrome (TLS) with various types of malignant tumor [6–8].

As rasburicase is a recombinant enzyme that does not naturally exist in humans, antibody production occurs at a rate of approximately 2%–10% after administration [9, 10], and regulatory authorities in Japan (Pharmaceuticals and Medical Devices Agency), as well as the supplier (Sanofi K.K., Tokyo), do not recommend the re-administration for patients previously exposed owing to its unestablished safety. Although the frequency of anaphylaxis upon re-administration is reported to be 0%–6.2% [11, 12], there has only been one report of a patient with anaphylaxis upon the first administration of rasburicase [4], and there have been no case reports of fatalities due to anaphylaxis.

Herein, we report a patient with anaphylaxis following the initial administration of rasburicase leading to death. We have also reviewed relevant published reports.

## Case presentation

A 71-year-old Japanese female was admitted to the hematology unit of our hospital to undergo chemotherapy for newly diagnosed diffuse large B cell lymphoma (DLBCL).

**Fig. 1:**
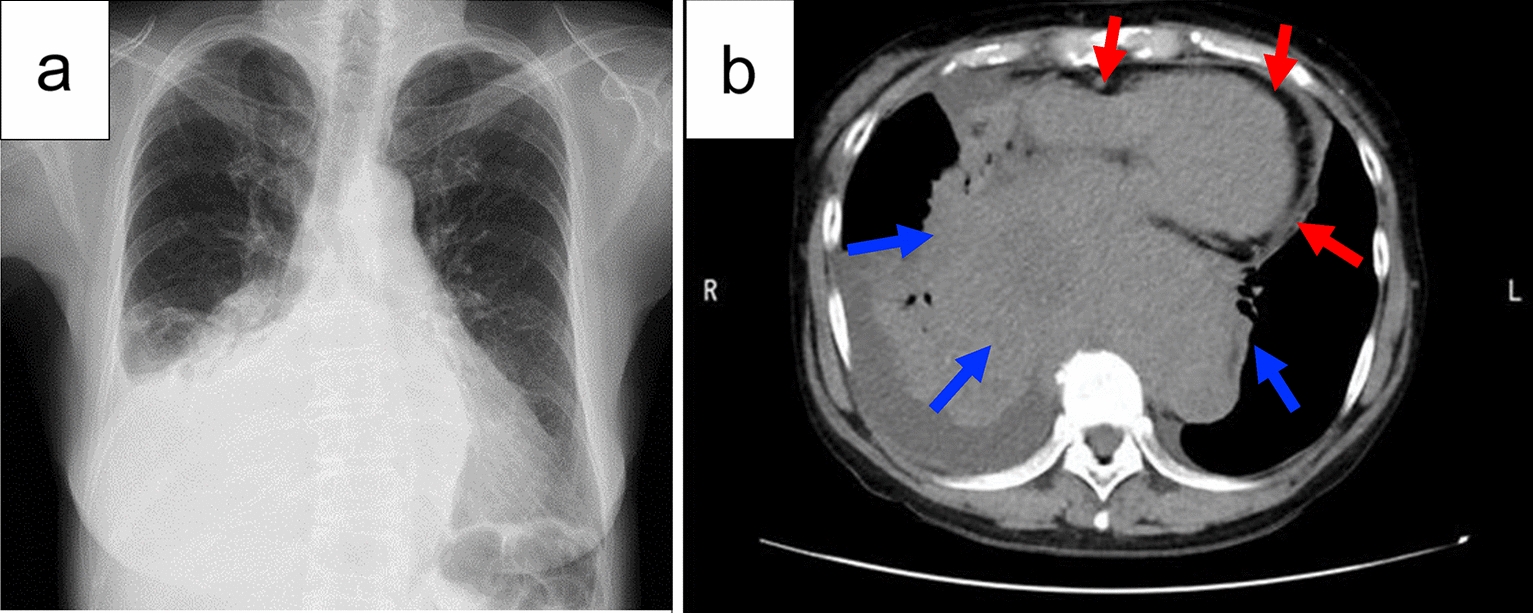
Chest X-ray and CT scan at diagnosis. **a** Chest X-ray showing atelectasis of the middle and lower lobes of the right lung. **b** Axial computed tomography showing a posterior mediastinal mass with a diameter of 12 cm (blue arrows) compressing the heart (red arrows). *CT* computed tomography

She had complained of dry cough and back pain that gradually worsened over 1 month. She consulted a local doctor and underwent a computed tomography (CT) scan, which revealed a posterior mediastinal mass with a diameter of 12 cm compressing the heart (Fig. 1). She underwent a bronchoscopic biopsy at our institution 10 days before her admission and was diagnosed with stage II DLBCL not otherwise specified, with a bulky mass. She had been undergoing treatment for bronchial asthma, taking steroids orally and via inhalation, and her condition was well controlled. She had a history of drug-induced rash with ambroxol and oral third-generation cephalosporin antibiotics. She had little to no impairment in her daily activities, with an Eastern Cooperative Oncology Group Performance Status of 1. On blood examination, lactate dehydrogenase and soluble interleukin-2 receptors showed mild elevation (379 U/L and 578.0 U/mL, respectively). All other parameters were within normal ranges.

**Fig. 2:**
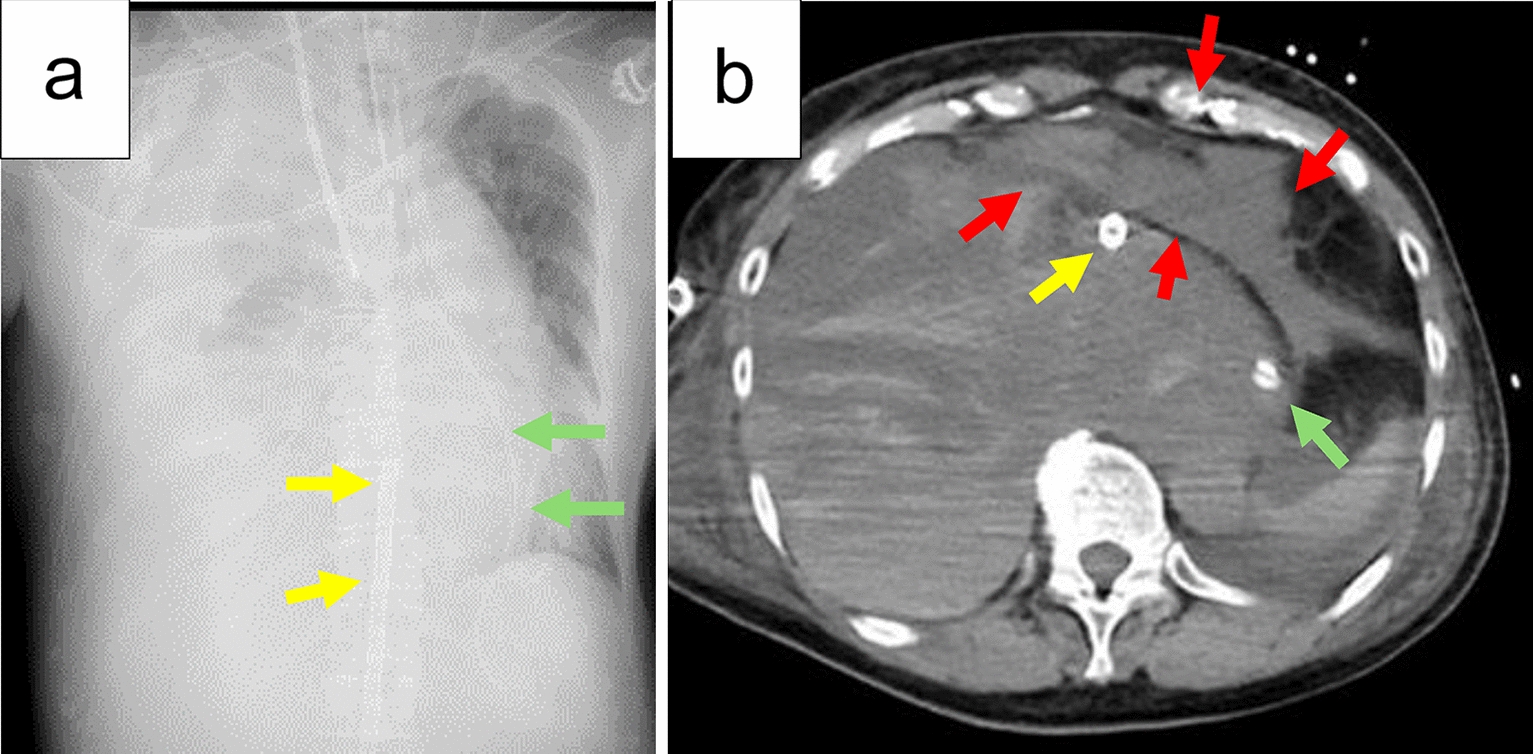
Chest X-ray and CT scan after resuscitation. **a** Chest X-ray showing atelectasis of the entire right lung and deviation of the mediastinum to the left. **b** Axial computed tomography showing massive fluid in the right pleural cavity. The heart had collapsed (red arrows). The yellow arrows show the devascularization catheter for percutaneous cardiopulmonary support in the inferior vena cava. The green arrows show the nasogastric tube in the esophagus. *CT* computed tomography

Although we planned to administer polatuzumab, rituximab, cyclophosphamide, doxorubicin, and prednisolone (Pola-R-CHP) as the initial therapy, we decided to administer rasburicase for the prevention of TLS prior to chemotherapy, considering the large tumor burden. Immediately after rasburicase administration, the patient complained of dyspnea, and generalized erythema and wheezing were observed. Shortly thereafter, her blood pressure plummeted and became unmeasurable, and she went into cardiopulmonary arrest (CPA). Immediate cardiopulmonary resuscitation (CPR) measures, such as chest compressions and artificial ventilation by ward physicians and nurses, were initiated. Pharmacological interventions, including adrenaline (1 mg of intramuscular followed by repetitive 1 mg of intravenous doses every 4–5 min), aggressive fluid resuscitation with 2 L of normal saline, steroids, and antihistamines, were also administered. However, it took 20 min to achieve recovery of the self-circulation. Subsequently, CPA occurred again and recovery of the self-circulation was achieved; however, the patient remained extremely unstable, necessitating the placement of a percutaneous cardiopulmonary support device. CT revealed massive pleural effusion compressing the right lung and the heart (Fig. 2). The patient died 5 days after the administration of rasburicase despite maximal supportive care.

**Fig. 3:**
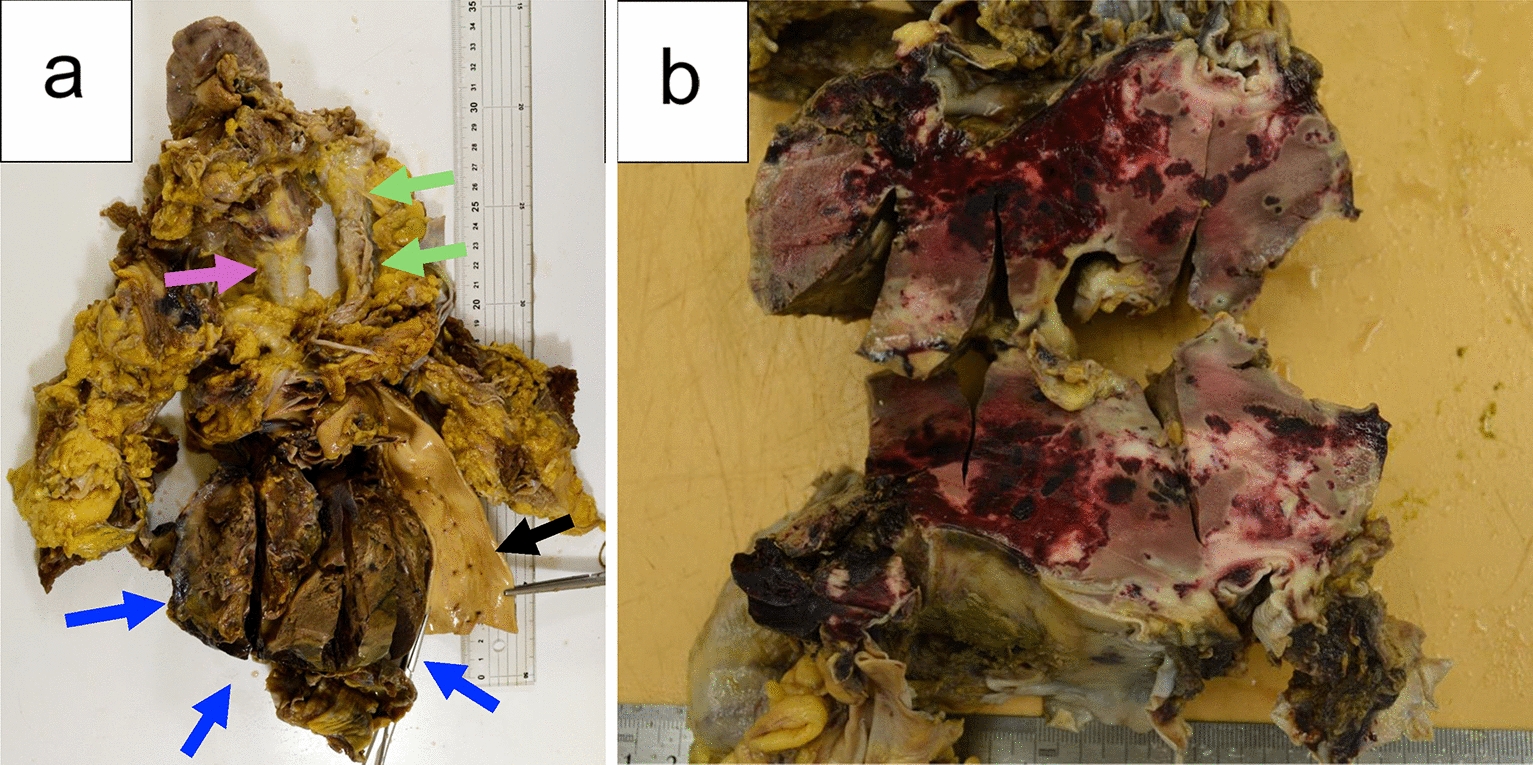
Gross pathological findings of the mediastinum. **a** The blue arrows show the crushed lymphoma in the posterior mediastinum. The green and purple arrows show the esophagus and the trachea, respectively. The black arrow shows the descending aorta. **b** Enlarged tumor section. Blood stains are visible on the surface of the crushed wound

Postmortem pathological examination revealed massive hemothorax filling the right pleural cavity and crushed lymphoma of the posterior mediastinum, which was speculated to have resulted from major bleeding due to mechanical injury of the tumor by chest compressions during CPR (Fig. 3). The right lung and heart had collapsed due to hemothorax.

## Discussion and conclusions

Anaphylaxis caused by rasburicase is rare, with past reports suggesting a rate of 0%–6.2% in the setting of re-administration [8, 9], and only one case of anaphylaxis has been described upon initial administration of rasburicase [4]. The estimated lifetime prevalence of anaphylaxis, regardless of the cause, is 0.3%–5.1% [13], and its mortality is estimated at 0.5–1 per million population [14]. The mortality rate of patients diagnosed with anaphylaxis is estimated at 0.2%–2.5% [15, 16]. Although these epidemiological data must be considered in light of information bias, it is undeniable that fatal anaphylaxis following the initial administration of rasburicase is extremely rare. In the present case, despite an immediate diagnosis of anaphylaxis and immediate care from the medical staff, a fatal outcome could not be avoided, probably in large part due to the presence of the large mediastinal maas, which contributed significantly to the circulatory shock.

**Fig. 4:**
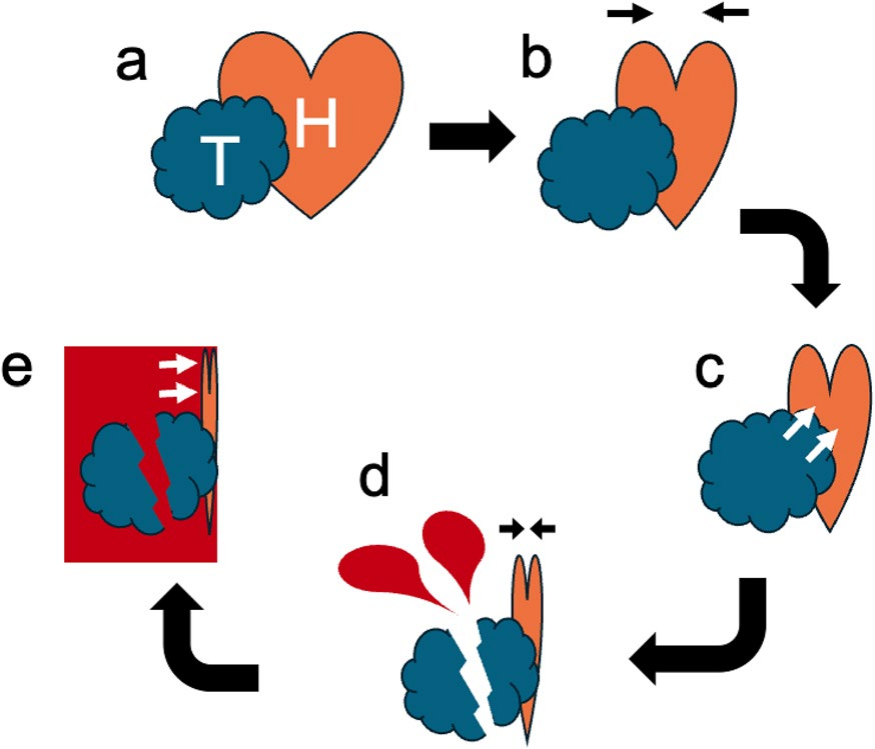
Schematic diagram of the cascade of shock and cardiopulmonary collapse. **a** *T* tumor, *H* heart (cardiovascular system). **b** Distributive shock mediated by anaphylaxis. **c** Obstructive shock due to defeating intracardial pressure by mechanical compression of the tumor. **d** Hypovolemic shock due to bleeding from the tumor. **e** Reinforced obstructive shock by tension hemothorax

The cardiopulmonary pathophysiology of severe anaphylaxis involves a combination of loss of intravascular volume due to increased vascular permeability, hypotension due to vasodilation, myocardial depression, and bradycardia, resulting in cardiovascular collapse, otherwise known as distributive shock [17]. In this case, a schematic diagram of the shock cascade triggered by anaphylaxis is shown in Fig. 4. The loss of intracardiac pressure due to distributive shock (Fig. 4b) attracted obstructive shock by mechanical compression of the tumor (Fig. 4c). Furthermore, massive bleeding from the crushed tumor lead to hypovolemic shock (Fig. 4d) followed by tension hemothorax (Fig. 4e), finally resulted in irreversible cardiopulmonary collapse.

Although there are no comprehensive reports describing the relationship between drug-induced anaphylaxis and asthma, most past clinical trials administering rasburicase have excluded patients with an apparent history of asthma from the study cohort [2–4, 9, 10, 18, 19]. Although there is no literature concerning rasburicase and asthma, Allen reported that one of six patients who developed anaphylaxis after repeated courses of rasburicase had comorbid asthma, which indicated that anaphylaxis did not occur at the initial administration [11]. Even though the safety of rasburicase in patients with asthma is not well established, in our case, it is likely that the mediastinal tumor, rather than asthma, played a more significant role in the fatal outcome. We do not believe that rasburicase should be contraindicated in all patients with an allergic predisposition given the very low risk of anaphylaxis and the benefits of preventing TLS. The appropriateness of rasburicase should be thoroughly evaluated, considering both the risk of TLS and the patient’s underlying risks.

The mechanism behind anaphylaxis to rasburicase on first exposure remains uncertain. There are reports of anaphylaxis on hepatitis B vaccination attributed to the protein derived from *S. cerevisiae*, the yeast used in hepatitis B vaccine and rasburicase synthesis [20]. Although the patient had no history of hepatitis B vaccination, it is possible that the patient was sensitized to this yeast. Non-IgE-mediated anaphylaxis can also occur with certain drugs on first exposure through either complement activation, MRGPRX-2 activation on mast cells, or, possibly, IgG-mediated anaphylaxis [21]. However, none of these mechanisms have been implicated in rasburicase-induced anaphylaxis.

In conclusion, although extremely rare, clinicians should consider the possibility of anaphylaxis, even with the initial administration of rasburicase. Anaphylaxis can lead to lethal outcomes when unfavorable conditions overlap.

## Data Availability

No datasets were generated or analysed during the current study.

## Abbreviations

CPA: Cardiopulmonary arrest
CPR: Cardiopulmonary resuscitation
CT: Computed tomography
DLBCL: Diffuse large B cell lymphoma
TLS: Tumor lysis syndrome
